# Development of a Novel Method for *in vivo* Determination of Activation Energy of Glucose Transport Across *S. cerevisiae* Cellular Membranes. A Biosensor-like Approach

**DOI:** 10.3390/s90301599

**Published:** 2009-03-09

**Authors:** Diego J. Kormes, Eduardo Cortón

**Affiliations:** 1 Biosensors and Bioanalysis Group, FCEN, UBA / Department of Biochemistry, Facultad de Ciencias Exactas y Naturales, Universidad de Buenos Aires, Ciudad Universitaria, Pab. 2, (1428) Buenos Aires, Argentina; E-Mail: diegok@gmail.com; 2 Biosensors and Bioanalysis Group, FCEN, UBA and CONICET / Department of Biochemistry, Facultad de Ciencias Exactas y Naturales, Universidad de Buenos Aires, Ciudad Universitaria, Pab. 2, (1428) Buenos Aires, Argentina

**Keywords:** Biosensors, microbial metabolism, Arrhenius plot, activation energy, *Saccharomyces cerevisiae*, glucose transport

## Abstract

Whereas biosensors have been usually proposed as analytical tools, used to investigate the surrounding media pursuing an analytical answer, we have used a biosensor-like device to characterize the microbial cells immobilized on it. We have studied the kinetics of transport and degradation of glucose at different concentrations and temperatures. When glucose concentrations of 15 and 1.5 mM were assayed, calculated activation energies were 25.2 and 18.4 kcal mol^−1^, respectively, in good agreement with previously published data. The opportunity and convenience of using Arrhenius plots to estimate the activation energy in metabolic-related processes is also discussed.

## 1. Introduction

Cell-based biosensors have been proposed for the determination of a large amount of substances or group of substances [1–4], although commercial available devices are only slowly reaching full maturity, and some of them, eventually, the market. Microbial biosensors have received less attention than enzymatic ones; one of the reasons explaining the prevalence of enzymatic biosensors could be the (frequently) low reproducibility obtained when microbial biosensors are used. Reproducibility is usually hard to improve, given the intrinsic high variability of living cells, and their capacity to adapt and show different metabolic pathways, by differential expression of inducible and repressible genes. Another problem is the sluggish response time, (given the necessary transport and metabolic processes involved) seriously restraining the maximum number of samples h^−1^ than a microbial biosensor could be able to analyze. Additionally, the specificity of microbial biosensors is usually low when compared with enzymatic ones.

Perhaps the major analytical application of microbial biosensors actually on the market are the biochemical oxygen demand biosensors (BOD_5_). This parameter estimates the amount of easily degradable organic material in water, by quantitative measurement of the respiration (oxygen consumption) of the microbial aerobic aquatic community present. Increased BOD_5_ values are indicative of organic pollution, by domestic or other organic-rich wastewater. Whereas the classical standard method needs five days of incubation to produce the analytical answer, biosensors can generate a more or less equivalent analytical parameter, usually named BOD_st_ (short-time BOD). Some BOD_st_ commercial devices are able to deliver the analytical data in less than one hour, dramatically improving the applicability of the BOD method. In addition, microbial biosensors could be used to evaluate the toxic effect of substances able to interfere in the respiratory or other metabolic microbial activity; in a recent work, the inhibitory effect of a number of antibiotics was assayed [5].

Here we introduce a different approach, using a *Saccharomyces cerevisiae* yeast based biosensor-like device, whereby the device is used to characterize metabolic attributes of the microbial material immobilized on it. By using the biosensor-like device presented here, we calculate the velocity of transport and degradation of glucose by *S. cerevisiae* at different temperatures and glucose concentrations; later, by the construction of Arrhenius plots (and assuming membrane transport as limiting step, as discussed later in this paper) the activation energy of glucose cellular membrane transport was estimated. We choose a *S. cerevisiae* strain as a microbial model to study the biosensor–like performance considering the large amount of information available about its metabolic characteristics.

Transport across the microbial cellular membrane is the first, obligatory step of hexose utilization; this process occurs generally by means of a carrier associated with the membrane, because the lipidic nature of the cellular membrane makes it impermeable to sugars. These carriers are similar to enzymes in some aspects; they are proteins, bond with their “substrates” in reversible complexes, and have a variable degree of specificity. Many of them are inducible or repressible proteins under direct genetic control, and show saturation kinetics. This kinetic behavior in the carriers can be explained because transportation takes place *via* a limited number of transporter proteins, rather than throughout the phospholipid bilayer; consequently, there is a maximum transport rate, *V_max_*, that is achieved only when the concentration gradient across the membrane is very large [6]. In yeast cells, a large family of related transporter proteins mediates the uptake of hexoses. In *S. cerevisiae,* the genes of 20 different hexose transporter-related proteins have been identified [7].

*S. cerevisiae* cells used in this biosensor-like device are kept under nutrient limitation conditions. Under these conditions, the respiratory activity is minimal, only the indispensable to guarantee the cell survival. This respiratory level, measured as CO_2_ production in starvation, can be used to define a baseline. When a suitable carbon source is added, some of it is taken up by the cells and degraded. Subsequently, the CO_2_ production, which is directly related with the hexose intake velocity (at a given concentration range), increases.

In an earlier work, we demonstrated the possibility of using this biosensor-like device as a rapid method to estimate apparent *K*_m_ of several carbohydrates membrane carriers, also, constructive details of the device presented and set-up were described [8]. Other ion-selective potentiometric electrodes (K^+^) have been used previously, to evaluate cellular membrane K^+^ efflux [9]; new instrumental methods allowing simplified procedures and *in vivo* measurements are required; *S. cerevisiae* based microbial biosensors fulfill both conditions and are an active area of scientific and technological research [10].

In this contribution, the determination of glucose-transport temperature dependence, at two glucose concentrations, was easily determined. The proposed method is compared with the data obtained using standard methods for membrane transport studies, which involves usually the incubation with a non-utilizable radioactively labeled sugar analog. The obtained data, presented as Arrhenius plots, allowed the rapid and cost effective estimation of the activation energy for glucose, giving information about the nature of membrane glucose transport.

## 2. Results and Discussion

The maximum slope, (rate, ΔmV min^−1^) data was transformed applying our CO_2_-electrode calibration curve (and considering Nernst sensibility of the potentiometric electrode at each temperature) to Δ[CO_2_] mol min^−1^ [11]. The use of CO_2_ molar concentration instead that mV data was necessary to obtain meaningful information, taken into account that the potentiometric electrode response is not lineal with CO_2_ concentration, and because the basal respiratory rate for *S. cerevisiae* ([CO_2_] at initial conditions, when glucose concentration in the media is negligible) is influenced strongly with temperature.

**Figure 1.**
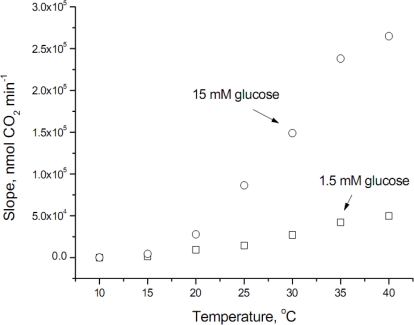
Variation of the maximum respiration rate (carbon dioxide production) at different temperatures and glucose concentrations. Each point represents the average from two independent experiments.

The raw data obtained at the temperature range studied is shown in Figure 1, where it can be observed that glucose transport at temperatures lower than 20°C was modest (values at 10°C were 49.8 ± 8.3 and 226.3 ± 31.9 nmol CO_2_ min^−1^ for 1.5 and 15 mM glucose, respectively). In addition, the velocity of glucose transport at 40°C does not follow an exponential increase, fact that can be explained because the optimum temperature for *S. cerevisiae* is around 34°C, therefore at the highest temperature assayed some inhibitory effect occurs.

**Figure 2.**
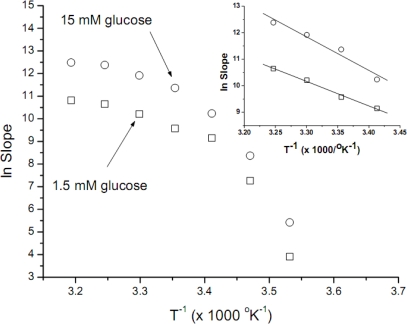
Arrhenius plots of the data presented in Figure 1. When data obtained at optimal and sub-optimal temperatures was used (inset), quasi-linear plots at both glucose concentrations assayed were obtained.

The data obtained was used to construct Arrhenius plots (Figure 2). These plots are not linear, thus being unuseful for further *E*_a_ calculations; convex Arrhenius plots have been obtained when other biological systems were assayed. Using as rationale the considerations expressed in the previous paragraph, the data obtained between 20 and 35°C was now used to construct new Arrhenius plots (Figure 2, inset). When these data was used, good linear correlations were obtained, with *r*-values of −0.997 and −0.978 for 1.5 mM and 15 mM of glucose, respectively. The *E*_a_ values calculated for both glucose concentrations (1.5 and 15 mM) were similar, 18.4 (±1.1) y 25.2 (±3.6) kcal mol^−1^ respectively. The uncertainty of the *E*_a_ values presented (SD values, also denominated standard error, Origin Pro 7.5) were estimated from the slopes of the curves with a standard error resulting from the error of slopes (−*E*_a_ *R*^−1^). In all cases the slopes were very significantly different from a zero slope. The error was first expressed as percentage of the slope (5.75 and 14.89 %, for 1.5 and 15 mM, respectively), afterward we assigned all the uncertainty (taken in account *R* is a constant) to *E*_a_ (1.06 and 3.63 kcal mol^−1^ for 1.5 and 15 mM, respectively).

When a fermentable carbon source was added to the buffer media, the yeast cells, previously in a starved situation, begin to transport and degrade the carbon source, a process that boosts the rate of CO_2_ production. The increase will correspond to the rate-limiting step, which according to some models of microbial metabolism, is the transport across the microbial membrane [12]. It must be noted that in the conditions used (nutrient and space limitation) no perceptible microbial growth is expected, confirmed by CFUs counting from the biosensor-like assembly after 24 h.

In order to estimate the *E*_a_, we plot the data obtained at temperatures not far away from *S. cerevisia*e yeast optimal ones, i.e., 20 to 35°C. These Arrhenius plots are shown in Figure 2, inset; it is interesting to point out that the *E*_a_ calculated for both glucose concentrations (1.5 and 15 mM) were similar, in the 20 kcal mol^−1^ range, consistent with values expected for membrane carriers, indicating also that the process involving transport across the cellular membrane (for the two glucose concentrations assayed) is driven by the same phenomena. Other authors have presented *E*_a_ data for glucose membrane transporters at concentrations of 0.2 and 20 mM in *Helicobacter pylori* cells [13], by radioactive tracer analysis techniques and by means of an analogue of d-glucose; their results suggest two different transporters with very different *E*_a_ values, of 1.6 and 12.2 kcal mol^−1^, respectively. The lower value is explained by transportation through membrane pores, whereas the higher value is associated to carrier-mediated facilitated diffusion; these passive transport systems are widespread among yeast.

Reinhardt *et al*. [14] have found an *E*_a_ of ≈ 15 kcal mol^−1^ for glucose transport in *S. cerevisiae*, describing also a pore system with lower *E*_a_. Our *E*_a_ data can be compared with the ones previously reported [13,14], providing evidence that glucose intake is mediated by protein transporters (carrier-mediated facilitated diffusion) at both glucose concentrations. The differences between the activation energies measured are similar, suggesting that the same type (or types) of carrier protein is involved in the transport. The good correlation between the data other authors obtained using accepted methods and the biosensor-like device method support the conclusion that this very simple method can be used, at least for gathering preliminary or exploratory information.

Although the *E*_a_ values obtained after the explained criteria used to reject some temperature data, are consistent with the published ones, it is a fact (Figure 2) that the overall shape of the Arrhenius plots we have obtained are convex. Similar plots have been obtained by microbiologists plotting the logarithm of growth rate constant against the reciprocal temperature, for several different microorganisms [15]. A continuous changing slope at different temperatures characterizes these Arrhenius plots; therefore, a poor fit is obtained if a linear model is chosen. Our data, obtained using non-growing cells produce very similar plots to those previously published using the growth rate of different microorganisms. Arrhenius’ law is of universal validity for elementary reactions; the failure to follow this model can be related to more complex metabolic responses, therefore the data obtained could be not exclusively correlated to membrane glucose transporters.

A more recent work [16] gives an alternative interpretation of the convex Arrhenius plots; it is pointed out that in some biological reactions convex plots have been observed, in a variety of thermostable and mesostable dehydrogenase and oxidase enzymes. This behavior could be interpreted as a lower rate than the expected rate at high temperatures, as probable in biological biocatalysts; further explanations for several biological systems are discussed in the mentioned paper. Moreover, a comparison between the Arrhenius analysis and the transition-state analysis of the temperature dependence of rate constants in biosensor studies have shown the relevance of the Arrhenius approach [17].

If the limiting step of the full respiratory process is assumed glucose transport across the cellular membrane, the obtained data, as we propose in this paper, can be directly related to glucose transport activation energy (*E*_a_). If transport across the cellular membrane were not the limiting step, the relation between the temperature and a more unspecific microbial respiratory activity (in non-growing cells) would be calculated using the experimental approach described here.

## 3. Experimental Section

### 3.1. Equipment

We used an in-house made CO_2_ electrode, based on the modification of a combined pH electrode; the calibration procedure was already described [10,18]. A low noise and high impedance instrumentation amplifier was used to adapt the signal from the electrode to the input of a 16 bit analogical to digital converter, after this the digital signal was acquired by a PC software.

### 3.2. Yeast Strain, Culture Media and Test Solutions

An industrial strain of *Saccharomyces cerevisiae* (baker’s yeast) was used as microbial model, as active dry yeast (SAF Argentine, Lesaffre group, Buenos Aires, Argentina). A rich media (YP) containing 10 g yeast extract, 10 g peptone and 10 g of glucose per liter was used for aerobic culture, cells were harvest by centrifugation at 5000 × *g* in a refrigerated centrifuge (4°C), 15 min. CFUs were counted using standard methods by serial dilutions and agar-plate counting. Phosphate buffer, 50 mM, pH 7.0 was used between experiments; when the biosensor-like device is dipped in, yeast consume rapidly the sugars previously transported into the cytoplasm, reaching an starved condition; unless otherwise stated we refer to this solution as buffer.

### 3.3. Preparation of Cellular Membranes and Biosensor-like Device Construction

The biosensor-like device was constructed by entrapment of *S. cerevisiae* cells on cellulose nitrate filtration membranes, 0.45 μm pore size; 5 mg of cells (dry weight) per cm^2^ of membrane were used. The cells were suspended in a drop of buffer solution and evenly spread onto the membrane on a circular area of about one cm^2^, which was the effective size of the electrode CO_2_ permeable silicone membrane. The membrane was tightly adjusted over the carbon dioxide electrode silicon membrane by an “O” Ring [8]. As the CO_2_ potentiometric electrode dioxide permeable membrane area in contact with the yeast cells is about 0.8 cm^2^, the estimated microbial active layer of our biosensor-like device was ≅ 4 mg.

### 3.4. Measuring System and Procedure

Measurements were performed in a 50 mL beaker, in phosphate buffer, at given temperatures; a constant flow of air was bubbled through the buffer, at 60 mL min^−1^. Following a stabilization time of at least three hours, the experiments were performed by adding glucose to the buffer solution, at given temperatures.

Once the CO_2_ values measured by the biosensor-like device became stabilized (the device was first dipped at 10°C in plain buffer), glucose was added (final concentration 1.5 mM) to the solution and the velocity of CO_2_ production was measured. Later, the device was washed and dipped in plain buffer, at the following assayed temperature (15°C); after stabilization, glucose was added (final concentration 1.5 mM), production of CO_2_ measured and washed; the procedure is repeated until reach the maximum assayed temperature. One different biosensor-like device was used for each glucose concentration assayed, and for each duplicate experiment. Given the high relationship between batch volume/mg yeast used (5 mg dry weight) the glucose concentration remains almost constant during the experiments. All experiments described here were done under these conditions, unless elsewhere noted.

The curves obtained shows a sharp increase in CO_2_ production, until they reach a new steady-state, corresponding to the new glucose concentration; the measurements and its interpretation was shown in our previously published paper [8].

### 3.5. Typical Recordings and Velocity Calculation

In order to estimate kinetic parameters, a velocity data must be used, which in this potentiometric system could be obtained from the maximum slope [8], directly in Δ*E* Δ*t*^−1^ or eventually, by the use of the carbon dioxide electrode calibration curve, in Δ[CO_2_] Δ*t*^−1^. The following procedure was used: *a*) The biosensor-like device was immersed in a baker with buffer (air bubbled), until the response becomes stabilized, defining a baseline; *b*) A concentrated glucose solution aliquot was added, 3–5 min are usually enough time to register the maximum slope (rate); *c*) The buffer-glucose solution was replaced by buffer solution (before rinsing twice the biosensor-like device with buffer); *d*) The response must return approximately to the values obtained in step *a* (baseline). The necessary time for that is typically 15–30 min, depending of the glucose concentration previously used and its exposure time, and *e*) Steps *b* to *d* are repeated at each temperature and glucose concentration assayed.

### 3.6. Temperature Dependence of Glucose Transport

The temperature dependence of glucose cellular membrane transport was measured using seven temperatures (10, 15, 20, 25, 30, 35 and 40°C) and two glucose concentrations (1.5 and 15 mM). Activation energies (*E*_a_) were calculated by solving the Arrhenius equation (Equation 1), expression that relate the effect of temperature over the rate constant *k*:

(1) $$k={\text{Ae}}^{-E_{a}/\mathrm{RT}}$$

where the preexponential factor A is assumed to be independent from temperature, *R* is the gas constant and *T* the absolute temperature. A more frequently used form of this equation is obtained by taking the natural logarithm, giving Equation 2 below:

(2) $$\text{ln}\;k=\text{ln}\;A-\frac{E_{a}}{\mathrm{RT}}\;\;,\;\text{or}\;\;\;\;\text{ln}\;k=-\frac{E_{a}}{\mathrm{RT}}+\text{constant}$$

The plot of ln *k* upon *T*^−1^ (known as Arrhenius plot) is usually a straight line, with a slope of −*E*_a_ *R*^−1^, providing the basis for the experimental determination of *E*_a_.

## 4. Conclusions

Briefly, we have established in this report that microbial-based biosensor-like devices can be successfully applied to study the *E*_a_ of glucose transport through the cellular membrane in *S. cerevisiae*. The limitations of the presented method to estimate *E*_a_ was discussed. Besides, similar biosensor-like devices could be successfully applied to carry out *in situ* and *in vivo* assays of enzymes within the cells, for example in permeabilized systems. The time required for the experiments are relatively short, mainly because the microbial cells are in close contact with the gas-permeable carbon dioxide sensor membrane; the microbial biosensors and biosensor-like devices possibilities are extremely broad, as an analytical method or even as a suitable method for rapid characterization of microorganisms [19].

The RSDs for the duplicates were relatively high (typically <20%), effort need to be done to improve the quality of the data obtained; the membrane yeast entrapment method used shows to be very influenced by operator skills, another more reproducible method should be employed.

As it was noted previously at Introduction, there are many different glucose transporters in *S. cerevisiae*; besides, its expression and relative proportion at cellular membrane is highly dependent of growing conditions and physiological state (8). Therefore, the *E*_a_ calculated here could be considered as a weighted average of the glucose transporters present at a given population of yeast cells. At the relatively high glucose concentration we used to grow *S. cerevisiae* (10 g L^−1^), is expected to be expressed mostly the denominated moderately low affinity carrier for glucose (20), with *K*_m_ values of about 10 mM for glucose. Yeast pre-treated at different conditions, including high and low glucose, ethanol growth and other conditions could be investigated using the method presented here. Evidently, in order to generate data corresponding to a single carrier, mutant strains expressing only one functional membrane transporter must be used.
